# Hypericin and hyperforin from St. John’s Wort are potent intestinal UGT inhibitors with distinct binding modes: mechanistic and quantitative prediction of clinical drug–drug interaction risk

**DOI:** 10.3389/fphar.2026.1899795

**Published:** 2026-09-10

**Authors:** Jinqian Chen, Yanlong Zhang, Xixi Zhao, Yating Cui, Tong Xu, Lingxia Liu, Zhenyu Zhao, Xichuan Li

**Affiliations:** 1 NHC Key Lab of Hormones and Development and Tianjin Key Lab of Metabolic Diseases, Tianjin Medical University Chu Hsien-I Memorial Hospital & Institute of Endocrinology, Tianjin, China; 2 Tianjin Medical College, Tianjin, China; 3 Tianjin Key Laboratory of Animal and Plant Resistance, College of Life Sciences, Tianjin Normal University, Tianjin, China

**Keywords:** drug–drug interaction, hyperforin, hypericin, intestinal metabolism, IVIVE, MM/PBSA, St. John’s wort, UDP-glucuronosyltransferase

## Abstract

St. John’s Wort (SJW; *Hypericum perforatum*) is widely used for mild-to-moderate depression and is known to induce certain cytochrome P450 enzymes (notably CYP3A4, with reported effects on CYP2C9 and CYP2C19) and intestinal P-glycoprotein, but its effects on UDP-glucuronosyltransferases (UGTs), the principal phase-II drug-metabolizing enzymes, remain largely uncharacterized. Using recombinant human UGTs with 4-methylumbelliferone as the probe substrate, we characterized inhibition by the two major SJW constituents, hypericin and hyperforin, across eleven isoforms, and combined AlphaFold2-based molecular docking, molecular dynamics, and MM/PBSA analyses with FDA 2020 static-model *in vitro–in vivo* extrapolation (IVIVE) to predict drug–drug interaction (DDI) risk. Both compounds were broad-spectrum UGT inhibitors, with hyperforin the most potent (UGT1A1 *K*_i_ = 1.46 μM; UGT2B7 *K*_i_ = 6.70 μM). On the clinically critical UGT1A1, hyperforin and hypericin occupied the aglycone pocket through two structurally distinct binding modes that both produced noncompetitive kinetics. Under a complete-dissolution assumption, IVIVE flagged hyperforin as a high-risk intestinal UGT inhibitor (*R*_1,gut_ = 69.49 for UGT1A1 and 15.93 for UGT2B7, far above the FDA threshold of 11); however, incorporating hyperforin’s poor (∼2%) dissolution and acid instability lowered the corrected *R*_1,gut_ to ∼2.0–2.9 (UGT1A1) and ∼1.2–1.4 (UGT2B7), indicating a potential rather than definitive intestinal interaction whose magnitude depends on the SJW formulation. These findings provide mechanistic and quantitative support for reported clinical SJW–drug interactions and highlight intestinal glucuronidation as a previously underappreciated SJW-mediated DDI pathway.

## 1 Introduction

**FIGURE 1:**
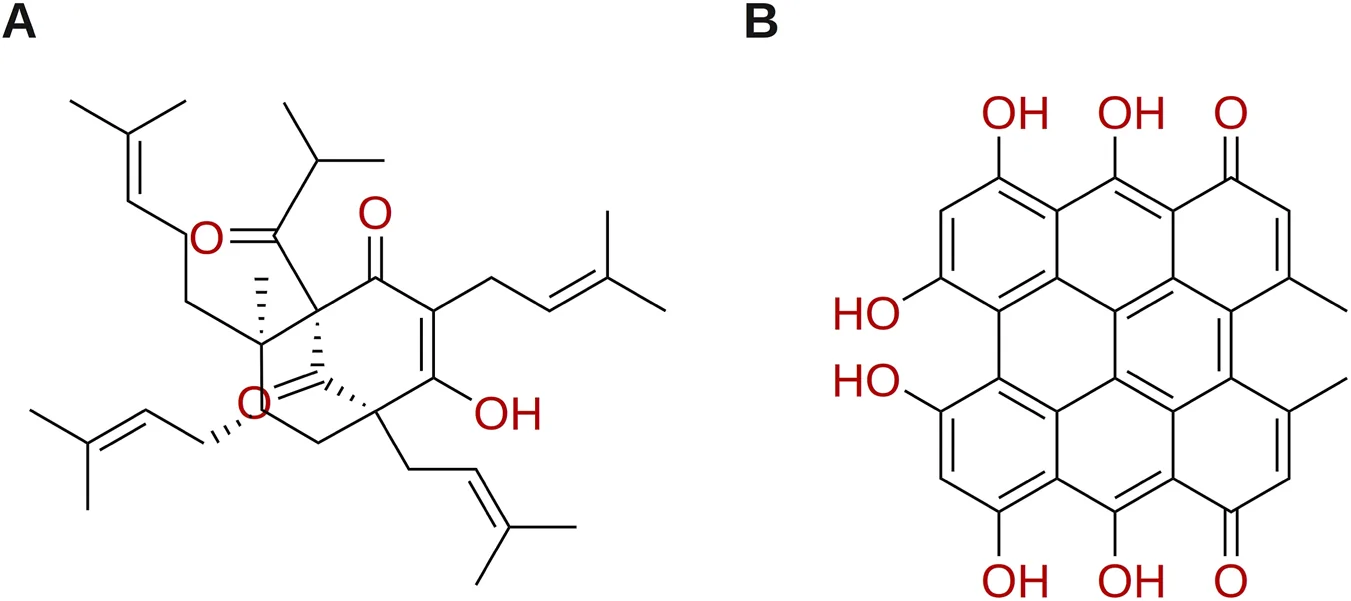
Chemical structures of the two major bioactive constituents of St. John’s Wort. **(A)** Hyperforin (CID 441298); **(B)** Hypericin (CID 3663). Oxygen-containing functional groups (carbonyl C=O; hydroxyl O–H) are highlighted in red.

Major depressive disorder (MDD) represents a significant global health burden (COVID-19 Mental Disorders Collaborators, 2021). St. John’s Wort (SJW), a medicinal herb, has gained prominence as an authorized treatment for mild-to-moderate depression in several countries, and its use has continued to increase in recent years. Standardized SJW extracts typically contain two key bioactive compounds: hypericin and hyperforin, which are believed to contribute to its antidepressant effects and have been studied for various other properties (Galeotti, 2017; Parvez and Rishi, 2019). Hyperforin (Figure 1A) is considered the primary driver of SJW’s antidepressant effects (Nicolussi et al., 2020) and also possesses anti-inflammatory, antiangiogenic, and anticancer properties (Li et al., 2023). Hypericin (Figure 1B), a phenanthroperylene compound, has also been reported to have potential antidepressant, anticancer, and antiviral activities (Karioti and Bilia, 2010; Liu. et al., 2020).

However, the widespread use of SJW raises significant clinical concerns regarding its potential for drug-drug interactions (DDIs) (Parvez and Rishi, 2019). It is well-documented that SJW alters the pharmacokinetics of numerous medications, including digoxin, warfarin, and oral contraceptives (Nicolussi et al., 2020), primarily by inducing cytochrome P450 (CYP) enzymes, such as CYP3A4, and the drug transporter P-glycoprotein (P-gp) (Chen et al., 2012). Hyperforin has been identified as the main constituent responsible for these interactions (Nicolussi et al., 2020).

Despite extensive research on SJW’s effects on phase I metabolism and drug transport, its impact on phase II metabolism—particularly the UDP-glucuronosyltransferase (UGT) enzyme family—remains largely unexplored. UGTs are responsible for the glucuronidation and clearance of approximately 35% of clinically used drugs, making them a crucial pathway for detoxification and elimination (Rowland et al., 2013). Among the major UGT isoforms, UGT1A1 is responsible for metabolizing approximately 15%–20% of clinical drugs subject to glucuronidation and is the sole enzyme responsible for bilirubin glucuronidation and the detoxification of the potent anticancer metabolite SN-38 (Barbarino et al., 2014). UGT2B7 is a critical enzyme for the glucuronidation of important drugs, including the opioid analgesic morphine and the antiretroviral agent zidovudine (Miley et al., 2007). Inhibition of these isoforms can lead to elevated drug exposure, reduced clearance, and potential toxicity. Given that patients taking SJW are often on other medications, understanding the herb’s effect on UGT-mediated metabolism is critical for ensuring patient safety.

Therefore, the objective of this study was to systematically investigate the DDI potential of SJW’s key constituents, hypericin and hyperforin, mediated through UGT inhibition. We hypothesized that hypericin and hyperforin, due to their lipophilic and polyphenolic structures, would exhibit significant inhibitory activity against major UGT isoforms, potentially representing an underappreciated mechanism of SJW-mediated DDIs. Specifically, this study aimed to: 1) determine the *in vitro* inhibitory effects and kinetic parameters of hypericin and hyperforin against a comprehensive panel of major human UGT isoforms; and 2) utilize these *in vitro* data, in conjunction with published human pharmacokinetic parameters, to quantitatively predict the clinical risk of hepatic and intestinal DDIs using IVIVE models based on FDA 2020 guidance (FDA, 2020). This research aims to fill a critical knowledge gap and provide a clear risk assessment to guide the safe co-administration of SJW with drugs metabolized by UGTs.

## 2 Materials and methods

### 2.1 Chemical reagents

Hyperforin (CAS 11079-53-1; catalog no. H911689, purity ≥98%) and hypericin (CAS 548-04-9; catalog no. H811167, purity ≥98%) were purchased from Macklin (Shanghai, China); both are chemically defined, purified single compounds rather than plant extracts. Recombinant human UGT isoforms (UGT1A1, UGT1A3, UGT1A6, UGT1A7, UGT1A8, UGT1A9, UGT1A10, UGT2B4, UGT2B7, UGT2B15, UGT2B17) were obtained from BD Gentest (Woburn, MA, USA). Uridine 5′-diphosphoglucuronic acid trisodium salt, 4-methylumbelliferone (4-MU), 4-methylumbelliferone-β-D-glucuronide, and 7-hydroxycoumarin were acquired from Sigma-Aldrich (St. Louis, MO, USA).

### 2.2 Screening for UGT inhibition

The inhibition of UGT activities was measured using recombinant human UGT isoforms with 4-methylumbelliferone (4-MU) as the probe substrate (Li et al., 2022; Xu et al., 2020). Incubation mixtures (200 μL total) contained recombinant UGT enzyme (0.1 mg/mL), 4-MU (100 μM, near reported *K*_m_), UDP-glucuronic acid (UDPGA, 5 mM), MgCl_2_ (5 mM), Tris-HCl buffer (50 mM, pH 7.4), and alamethicin (50 μg/mg protein, pre-incubated on ice for 15 min to ensure membrane permeabilization). Hypericin and hyperforin were dissolved in dimethyl sulfoxide (DMSO; final concentration ≤0.5% v/v, confirmed not to affect UGT activity in vehicle controls). Reactions were initiated by addition of UDPGA, incubated at 37 °C for 30–60 min (within the linear range determined in preliminary time-course experiments), and terminated by addition of an equal volume of ice-cold acetonitrile containing 7-hydroxycoumarin (internal standard). Although 4-MU is not isoform-selective, it is a well-established and widely used universal substrate for UGT activity screening, enabling direct cross-isoform comparisons under identical assay conditions. The use of isoform-specific substrates in future studies is warranted to validate these findings. HPLC analysis was performed using a Waters 2695 system with a C18 reverse-phase column (4.6 × 250 mm, 5 μm). The mobile phase consisted of acetonitrile (A) and 0.5% formic acid (B) with gradient: 0–5.5 min, 10%–65% A; 5.5–9 min, 65% A; 9–10.5 min, 65%–10% A; 10.5–12.5 min, 10% A; flow rate 1.0 mL/min; detection at 316 nm (4-MUG). All assays were performed in triplicate using two independent batches of recombinant enzyme.

**Positive controls and assay performance**. To confirm that the recombinant enzyme preparations were functionally active and inhibitable, positive-control inhibitors were included under the same incubation conditions. Atazanavir, a clinically characterized UGT1A1 inhibitor (*K*_i_ ≈ 1.9 μM (Zhang et al., 2005)), was tested against UGT1A1 (1 and 100 μM), and diclofenac, a well-established UGT2B7 inhibitor (Uchaipichat et al., 2004), was tested against UGT2B7 (100 μM); diclofenac was used as a positive-control inhibitor rather than an isoform-selective probe. Residual activity (%) was calculated as (4-MUG/internal-standard peak-area ratio in the presence of inhibitor) ÷ (ratio in the vehicle control) × 100. The signal-to-noise ratio of the 4-MUG peak far exceeded the acceptance criterion of 10:1 in all quantified samples (typically >1000:1). Positive-control measurements were performed in triplicate (n = 3) (Supplementary Figure S1).

### 2.3 Concentration-dependent inhibition

For UGT isoforms showing greater than 80% activity reduction in the preliminary screening, a concentration-dependent inhibition study was subsequently performed. The final concentrations of hypericin and hyperforin tested were 0, 0.5, 1, 5, 10, 20, 40, 50, 60, 80, and 100 μM. Data were plotted as concentration-inhibition curves, and IC_50_ values were calculated by nonlinear regression analysis using GraphPad Prism 8.0. It should be noted that IC_50_ values were determined at a single fixed substrate concentration and serve as rank-order screening indicators only; all IVIVE calculations (Section 2.5) rely exclusively on the mechanistic inhibition constant *K*_i,u_ derived from full kinetic analysis (Section 2.4).

### 2.4 Kinetic analysis

Inhibition kinetic parameters were determined for the most affected UGT isoforms based on IC_50_ results. Incubations were performed with four substrate concentrations (ranging from 1/5 × *K*_m_ to 5 × *K*_m_) in the absence and presence of four inhibitor concentrations chosen to span the IC_50_ value. The type of inhibition was ascertained by fitting velocity data to standard inhibition models, and the inhibition constant (*K*ᵢ) was calculated from the best-fit model. Lineweaver-Burk plots and secondary slope replots were used for graphical representation. *K*_i_ values were calculated by nonlinear regression using the following Equations 1–4 (Austin et al., 2002; Cheng and Prusoff, 1973; Cer et al., 2009; Buker et al., 2019):

$$\text{Competitive}:V=V_{\max}\left[S\right]/\;\left\{K_{m}\left(1+\left[I\right]/K_{i}\right)+\left[S\right]\right\}$$ (1)

$$\text{Noncompetitive}:V=V_{\max}\left[S\right]/\;\left\{\left(K_{m}+\left[S\right]\right)\left(1+\left[I\right]/K_{i}\right)\right\}$$ (2)

$$\text{Uncompetitive}:V=V_{\max}\left[S\right]/\;\left\{K_{m}+\left[S\right]\left(1+\left[I\right]/K_{i}\right)\right\}$$ (3)

$$\text{Mixed}:V=V_{\max}\left[S\right]/\;\left\{K_{m}\left(1+\left[I\right]/K_{i}\right)+\left[S\right]\left(1+\left[I\right]/\alpha K_{i}\right)\right\}$$ (4)

To correct for non-specific microsomal binding and reduce systematic bias, *K*_i_ was corrected by the unbound fraction in microsomal incubation (*f*_u,inc_) to yield *K*_i,u_. The *f*_u,inc_ values were estimated using an established empirical equation (Austin et al., 2002), which relates *f*_u,inc_ to logD_7_._4_ and microsomal protein concentration. Since both hypericin and hyperforin are acidic compounds (pK_a_ < 7.4), their respective logD_7_._4_ values were used in the calculation. The estimated *f*_u,inc_ values were 0.993 for hypericin (logD_7_._4_ ≈ 1.6) and 0.931 for hyperforin (logD_7_._4_ ≈ 4.5), consistent with low non-specific binding at the microsomal protein concentrations used in UGT assays. We acknowledge that experimental determination of fuinc (e.g., by rapid equilibrium dialysis) would provide greater accuracy, particularly for highly lipophilic compounds such as hyperforin (logD_7_._4_ ≈ 4.5). This represents a limitation of the current study.

### 2.5 Molecular docking simulations

To provide a structural basis for the observed inhibition kinetics, molecular docking simulations were performed against AlphaFold2-predicted structures of the six UGT isoforms that showed strong inhibition (UGT1A1, UGT1A6, UGT1A9, UGT2B4, UGT2B7, UGT2B15). Full-length monomeric structures were retrieved from the AlphaFold2 Protein Structure Database (Jumper et al., 2021) (UniProt accessions P22309, P19224, O60656, P06133, P16662, P54855) (Varadi et al., 2022). Structural confidence was assessed using per-residue pLDDT scores; mean pLDDT across the six isoforms ranged from 91.0 to 94.0, with 91.6%–96.2% of residues exceeding the confident threshold (pLDDT >70) and 78.2%–87.3% exceeding the very-high-confidence threshold (pLDDT >90) (Supplementary Table S2; Supplementary Figure S3). Binding pocket residues identified by PLIP analysis and MM/PBSA per-residue decomposition showed local pLDDT values of 88.5 (UGT1A1) and 92.2 (UGT2B7), indicating high local structural accuracy at ligand-binding regions. The characteristic low-confidence regions at the N-terminus (signal peptide, residues 1–25) and C-terminus (transmembrane anchor, residues ∼510–533) did not affect the cytosolic catalytic domain or the ligand-binding pocket analyzed in this study.

Receptor structures were prepared in AutoDockTools (Morris et al., 2009) by removing crystallographic waters, adding polar hydrogens, and assigning Kollman charges. Hypericin (CID 3663) and hyperforin (CID 441298) were retrieved from PubChem, energy-minimized, and assigned Gasteiger partial charges. Docking was performed with AutoDock Vina v1.2.0 (Trott and Olson, 2010) using a 30 × 30 × 30 Å search box centered on the N-terminal aglycone-binding region (residues 38–200), consistent with the established aglycone-binding domain assignment for UGTs (Locuson and Tracy, 2007; Magdalou et al., 2010). Exhaustiveness was set to 32 with 10 docking modes per ligand. The top-ranked pose (most negative ΔG) was used for downstream PLIP analysis (Adasme et al., 2021) and as the starting structure for molecular dynamics simulations.

### 2.6 Molecular dynamics simulations and block analysis

To characterize binding-mode stability and dynamic protein–ligand contacts, 100 ns molecular dynamics simulations were performed for three representative complexes: hypericin/UGT1A1, hyperforin/UGT1A1, and hypericin/UGT2B7. The starting complexes were the top-ranked docked poses from the validated docking pipeline. Simulations were performed using GROMACS 2022.2 (Abraham et al., 2015) with the AMBER ff14SB force field (Maier et al., 2015) for proteins and the General AMBER Force Field (GAFF2) (Wang et al., 2004) for ligands. Ligand topologies were generated using sobtop_1.0 with RESP charges derived at the HF/6-31G* level. Each system was solvated with TIP3P water in a cubic box (minimum 1.0 nm protein–box distance), neutralized with Na^+^/Cl^−^ ions, and brought to 0.15 M ionic strength. Energy minimization (steepest descent, ≤50,000 steps) was followed by 100 ps NVT equilibration (310 K, V-rescale thermostat) and 100 ps NPT equilibration (1 bar, Parrinello–Rahman barostat). Production simulations were 100 ns NPT (2 fs timestep, 10 ps output frequency); LINCS constrained bonds with hydrogens, PME handled long-range electrostatics (cutoff 1.0 nm), and van der Waals interactions used a 1.0 nm cutoff.

Trajectory analyses used built-in GROMACS tools: gmx rms (backbone RMSD), gmx gyrate (Rg), gmx sasa (solvent-accessible surface area), gmx hbond (intermolecular hydrogen bonds; 0.35 nm distance and 30° angle cutoffs), and gmx rmsf (per-residue fluctuations). The free-energy landscape was computed by projecting the trajectory onto the first two principal components (gmx covar/anaeig) and applying gmx sham. To assess single-trajectory convergence, block-averaging analysis was performed (Grossfield and Zuckerman, 2009): the equilibrated portion of each 100 ns trajectory (20–100 ns) was divided into four 20 ns blocks; block means and the inter-block standard deviation (SD) are reported as the principal uncertainty estimates. We note that intra-trajectory block analysis underestimates true simulation-to-simulation variance relative to independent replicate trajectories (Genheden et al., 2015), and the reported SDs should therefore be interpreted as indicators of intra-trajectory convergence.

### 2.7 MM/PBSA binding free energy calculations

Binding free energies were calculated using gmx_MMPBSA v1.6.4 (V et al., 2021; Hou et al., 2011) under the single-trajectory protocol. Frames were extracted at 800 ps intervals from the equilibrated portion of each 100 ns trajectory (20–100 ns), yielding 101 frames per system. The Poisson–Boltzmann solver implemented in sander (AMBER) was used with internal dielectric εin = 1.0, external dielectric εout = 80.0, ionic strength 0.15 M, and the LCPO non-polar surface area model. Per-residue energy decomposition (idecomp = 2) was performed for all residues within 5Å of the ligand. Statistical errors are reported as the sample standard deviation across the 101 frames.

Entropy contributions (−TΔS) were not explicitly calculated due to the prohibitive computational cost of normal-mode analysis for systems of this size and the known limitations of normal-mode entropy for highly flexible ligands (Genheden et al., 2015). Consequently, absolute ΔG_bind_ values represent enthalpy-dominated estimates and are not quantitatively compared to experimental *K*_i_-derived ΔG. Energy component patterns and per-residue contributions are instead used for mechanistic interpretation.

### 2.8 In vitro–in vivo extrapolation (IVIVE) of DDI risk

*In vivo* DDI potential was predicted using a static model based on FDA 2020 guidance (FDA, 2020). The maximum unbound plasma concentration ([I]_max,u_) was calculated from reported C_max_ values and a literature-based unbound fraction in plasma (*f*_u,p_). The intestinal inhibitor concentration ([I]_gut_) was calculated assuming complete dose dissolution in 250 mL of intestinal fluid, representing a theoretical upper-bound exposure. We note that this assumption does not account for the limited dissolution of hyperforin from commercial SJW formulations (reported as approximately 2% (Jürgenliemk and Nahrstedt, 2003)) or its acid instability under gastric pH conditions (Ang et al., 2004); a sensitivity analysis incorporating these physicochemical factors is presented in Section 3.6. Hepatic (*R*_1_) and intestinal (*R*_1,gut_) risk ratios were calculated as:

$$R_{1}=1+{\left[I\right]}_{\max,u}\;/\;K_{i,u}\;\left(\text{Systemic}\right)$$

$$R_{1}{,}_{\text{gut}}=1+{\left[I\right]}_{\text{gut}}\;/\;K_{i,u}\;\left(\text{Intestinal}\right)$$

Per FDA guidance, *R*_1_ ≥ 1.02 indicates potential for clinically significant systemic DDIs, and *R*_1,gut_ ≥ 11 indicates high potential for intestinal DDIs. Pharmacokinetic parameters were derived primarily from published pharmacokinetic datasets (Schulz et al., 2005; Biber et al., 1998). To assess prediction robustness, sensitivity analyses were conducted using a range of reported C_max_ values (approx. 80–300 ng/mL) (Schulz et al., 2005; Gödtel-Armbrust et al., 2007) and commercial dosage ranges (hyperforin: 9–17 mg/dose) (Gödtel-Armbrust et al., 2007). A more detailed AUC ratio-based IVIVE model was also applied and is described in the Supplementary Material.

The present study evaluated reversible inhibition only. Time-dependent (mechanism-based) inhibition was not assessed. Given hyperforin’s known CYP-inducing activity, future studies should investigate whether it also acts as a mechanism-based UGT inhibitor. This is acknowledged as a limitation of the current study.

### 2.9 Statistical analysis

All assays were performed as three independent biological replicates (n = 3; not technical replicates), each using two independent batches of recombinant enzyme. GraphPad Prism 8.0 software (version 8.0.1) was used to calculate IC_50_ and *K*ᵢ values with 95% confidence intervals (95% CI). Data are presented as mean ± S.D. Statistical significance was set at *p* < 0.05.

## 3 Results

### 3.1 Inhibition of UGT activities by hypericin and hyperforin

The inhibitory effects of hypericin and hyperforin were systematically evaluated against eleven human UGT isoforms (UGT1A1, UGT1A3, UGT1A6, UGT1A7, UGT1A8, UGT1A9, UGT1A10, UGT2B4, UGT2B7, UGT2B15, UGT2B17) using recombinant enzymes.

**FIGURE 2:**
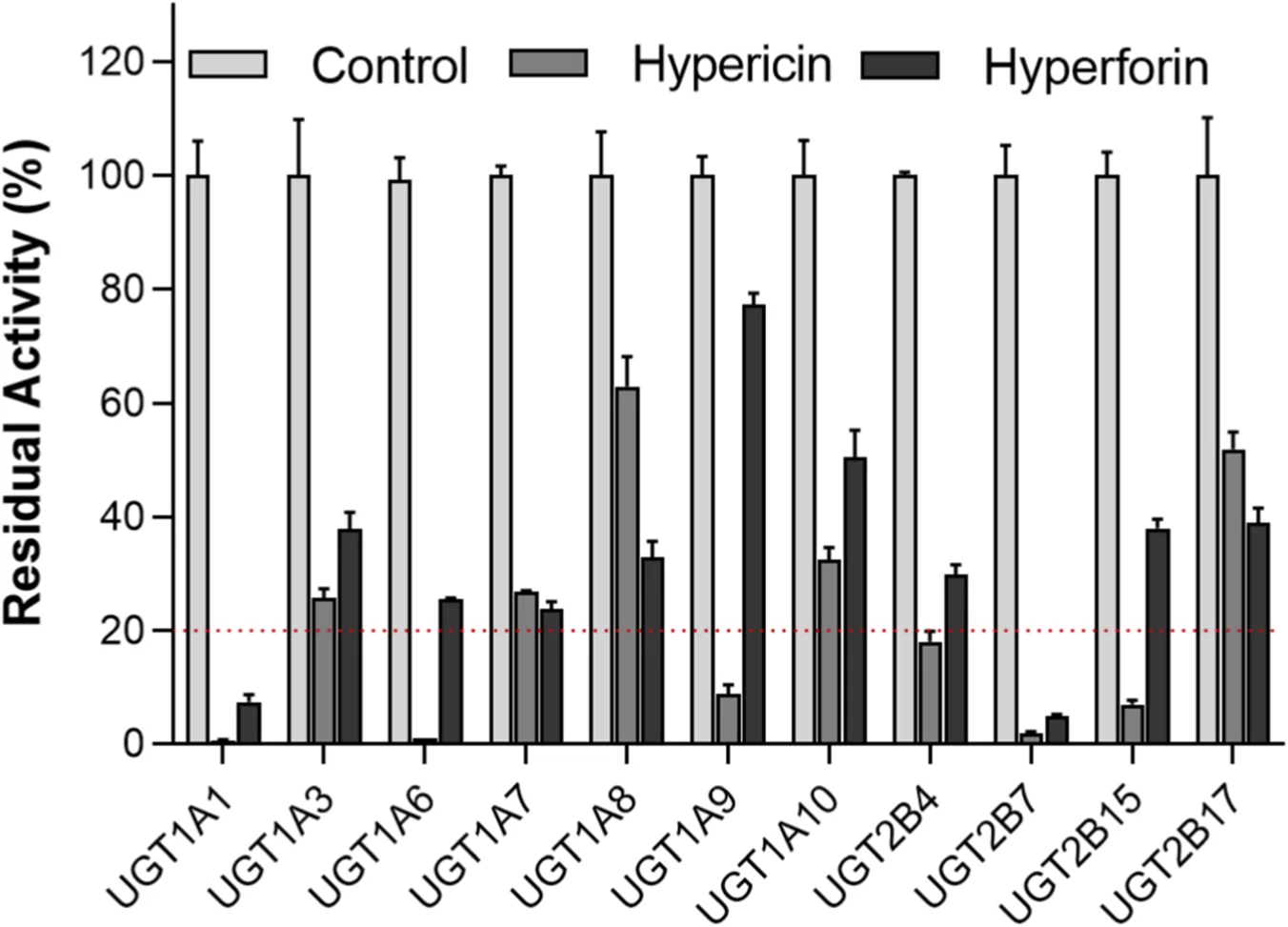
Primary screening of UGT inhibitory activity of hypericin and hyperforin across eleven human UGT isoforms at 100 μM. Residual 4-methylumbelliferone (4-MU) glucuronidation activity is expressed as a percentage of the vehicle control (mean ± S.D., n = 3). Red dotted line: 20% residual activity threshold. The “no-inhibitor” condition (100% activity) served as the vehicle (negative) control; positive-control inhibition data are shown in Supplementary Figure S1.

To identify specific UGT isoforms, both compounds were screened against a panel of 11 major recombinant human UGTs. At 100 μM, both compounds exhibited broad-spectrum inhibition, reducing 4-MU glucuronidation by over 50% for nearly all tested isoforms (Figure 2). The only exception was UGT1A8, which retained 53.51% activity in the presence of hypericin. Hypericin showed particularly robust inhibition (>80%) against six isoforms: UGT1A1, UGT1A6, UGT1A9, UGT2B4, UGT2B7, and UGT2B15, with inhibition ranging from 84.19% to 99.38%. Similarly, hyperforin potently inhibited UGT1A1 and UGT2B7 by 90.18% and 94.59%, respectively. These isoforms were selected for detailed kinetic analysis.

**FIGURE 3:**
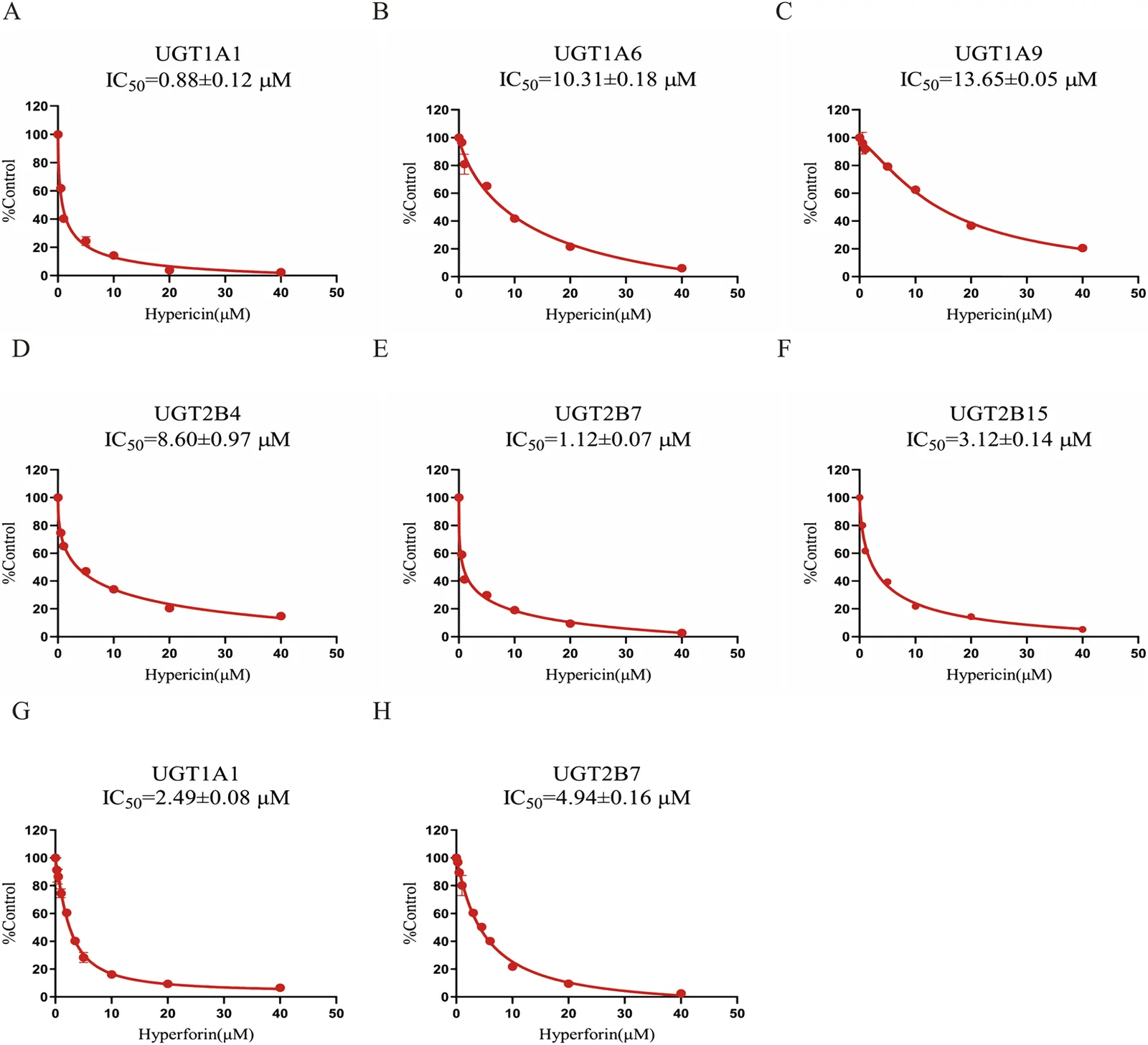
Concentration-inhibition curves for hypericin **(A–F)** and hyperforin **(G–H)** against selected UGT isoforms. IC_50_ values (mean ± S.D.) by nonlinear regression. **(A)** Hypericin/UGT1A1; **(B)** UGT1A6; **(C)** UGT1A9; **(D)** UGT2B4; **(E)** UGT2B7; **(F)** UGT2B15; **(G)** Hyperforin/UGT1A1; **(H)** UGT2B7, 4.94 ± 0.16 μM.

Concentration-inhibition curves were subsequently generated for the isoforms showing >80% inhibition (Figure 3). Hypericin potently inhibited UGT1A1, UGT2B4, UGT2B7, and UGT2B15, with IC_50_ values below 10 μM, while exhibiting moderate inhibition of UGT1A6 and UGT1A9 (IC_50_ values of 10–15 μM). Hyperforin showed potent inhibition of UGT1A1 (IC_50_ = 2.49 ± 0.08 μM) and UGT2B7 (IC_50_ = 4.94 ± 0.16 μM).

### 3.2 Kinetic analysis

**FIGURE 4:**
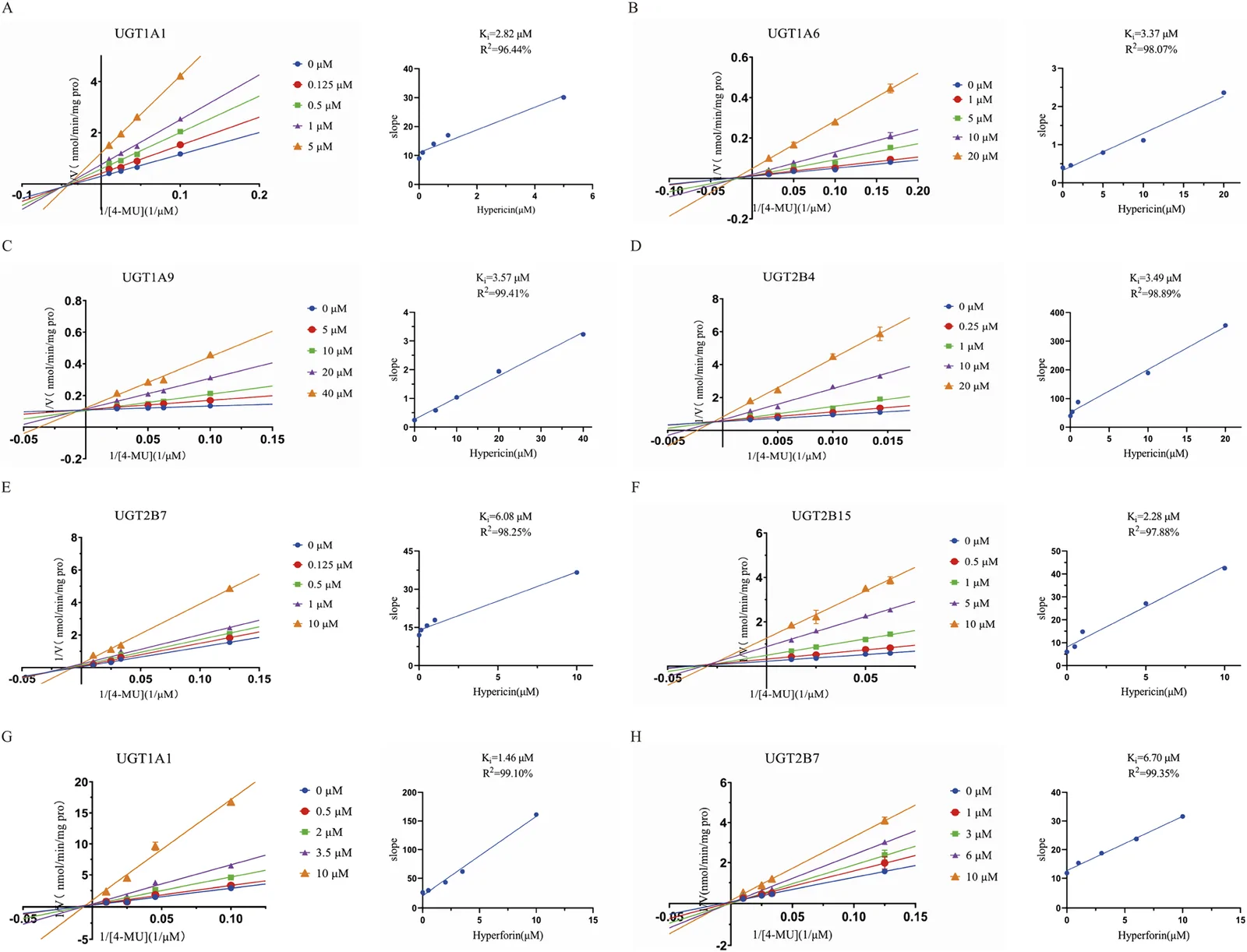
Inhibition kinetics of hypericin and hyperforin against selected human UGT isoforms. Lineweaver–Burk plots and secondary slope replots. Panels: **(A)** Hypericin/UGT1A1, noncompetitive, *K*_i_ = 2.82μM; **(B)** UGT1A6, noncompetitive, 3.37μM; **(C)** UGT1A9, competitive, 3.57μM; **(D)** UGT2B4, mixed (α = 3.09), 3.49μM; **(E)** UGT2B7, mixed (α = 0.13), 6.08μM; **(F)** UGT2B15, noncompetitive, 2.28μM; **(G)** Hyperforin/UGT1A1, noncompetitive, 1.46μM; **(H)** Hyperforin/UGT2B7, noncompetitive, 6.70 μM.

Enzyme kinetic experiments were conducted to elucidate the inhibitory mechanisms (Figure 4). Lineweaver-Burk plots and nonlinear regression analysis (Li et al., 2015) revealed multiple inhibition modes for hypericin: noncompetitive inhibition of UGT1A1, UGT1A6, and UGT2B15 (*K*ᵢ = 2.82, 3.37, and 2.28 μM, respectively); mixed inhibition of UGT2B4 (*K*ᵢ = 3.49 μM, α = 3.09) and UGT2B7 (*K*ᵢ = 6.08 μM, α = 0.13); and competitive inhibition of UGT1A9 (*K*ᵢ = 3.57 μM). Hyperforin acted as a noncompetitive inhibitor of both UGT1A1 (*K*ᵢ = 1.46 μM) and UGT2B7 (*K*ᵢ = 6.70 μM). The unbound inhibition constants (*K*_i_,_u_) used for the IC_50_ and *K*_i_ values for each inhibitor–isoform pair are given in Table 1; the unbound inhibition constants (*K*_i,u_) used for the IVIVE analysis are listed in Table 2.

**TABLE 1:** Inhibition kinetic parameters (IC_50_ and *K*ᵢ) and inhibition type of hypericin and hyperforin on human UGT isoforms.

​	Hypericin			Hyperforin		
UGTs	IC_50_ (μM)	*K*ᵢ (μM)	Inhibition type	IC_50_ (μM)	*K*ᵢ (μM)	Inhibition type
UGT1A1	0.88 ± 0.12	2.82	Noncompetitive	2.49 ± 0.08	1.46	Noncompetitive
UGT1A6	10.31 ± 0.18	3.37	Noncompetitive	–	–	–
UGT1A9	13.65 ± 0.05	3.57	Competitive	–	–	–
UGT2B4	8.60 ± 0.97	3.49 (α = 3.09)	Mixed	–	–	–
UGT2B7	1.12 ± 0.07	6.08 (α = 0.13)	Mixed	4.94 ± 0.16	6.70	Noncompetitive
UGT2B15	3.12 ± 0.14	2.28	Noncompetitive	–	–	–

Values are mean ± S.D., from three independent experiments. *K*ᵢ values from global nonlinear regression. α: cooperativity factor for mixed-inhibition (UGT2B4 and UGT2B7 for hypericin). –: not tested (inhibition <80% at 100 μM in primary screen).

### 3.3 Molecular docking analysis

To rationalize the inhibition kinetics at the atomic level, molecular docking simulations were performed for hypericin against the six strongly inhibited UGT isoforms (UGT1A1, UGT1A6, UGT1A9, UGT2B4, UGT2B7, UGT2B15) and for hyperforin against UGT1A1 and UGT2B7. All docked poses localized to the N-terminal aglycone-binding pocket, consistent with the established aglycone-binding domain assignment for UGTs (Locuson and Tracy, 2007; Magdalou et al., 2010). Predicted binding energies for hypericin across the six tested isoforms ranged from −10.2 to −11.3 kcal/mol (Supplementary Table S3). Hyperforin showed somewhat less favorable scores (ΔG_dock_ = −8.4 kcal/mol for UGT1A1 and −7.1 kcal/mol for UGT2B7), yet directionally consistent with the experimentally measured *K*_i_ values (1.46 μM and 6.70 μM, respectively). PLIP analysis (Supplementary Table S3) revealed that hyperforin engaged a compact set of hydrophobic residues within the UGT1A1 aglycone pocket (PHE92, LEU95, ILE116, THR112, PHE394), whereas hypericin established broader contacts spanning the aglycone pocket (ILE116, PHE153) together with envelope-helix (ALA174, PRO176), N-terminal (SER38, HIS39), and C-terminal (ASP396) residues.

To validate the docking pipeline, cross-docking benchmarking was performed using atazanavir, a clinically characterized UGT1A1 inhibitor (*K*_i_ = 1.9 μM, mixed-type (Zhang et al., 2005)). The top-ranked pose yielded ΔG_dock_ = −9.3 kcal/mol, placing it within ∼1.5 kcal/mol of the experimentally derived free energy (**≈**−7.8 kcal/mol from *K*_i_ = 1.9 μM) once the systematic Vina overestimation for large flexible ligands is taken into account. The pose settled into the N-terminal aglycone-binding pocket, where it made hydrophobic contacts with PHE92, LEU95, ILE116, LEU123, PHE153, ALA174, PRO176, and PRO196; three hydrogen bonds anchored to SER38, ARG257, and ASP396; and π–π stacking against PHE92. This contact profile overlaps substantially with the hyperforin binding signature recovered by MM/PBSA per-residue decomposition (see Section 3.6), confirming that the pipeline reliably reproduces the canonical UGT1A1 aglycone-pocket binding mode.

### 3.4 Molecular dynamics simulations of UGT–Ligand complexes

**FIGURE 5:**
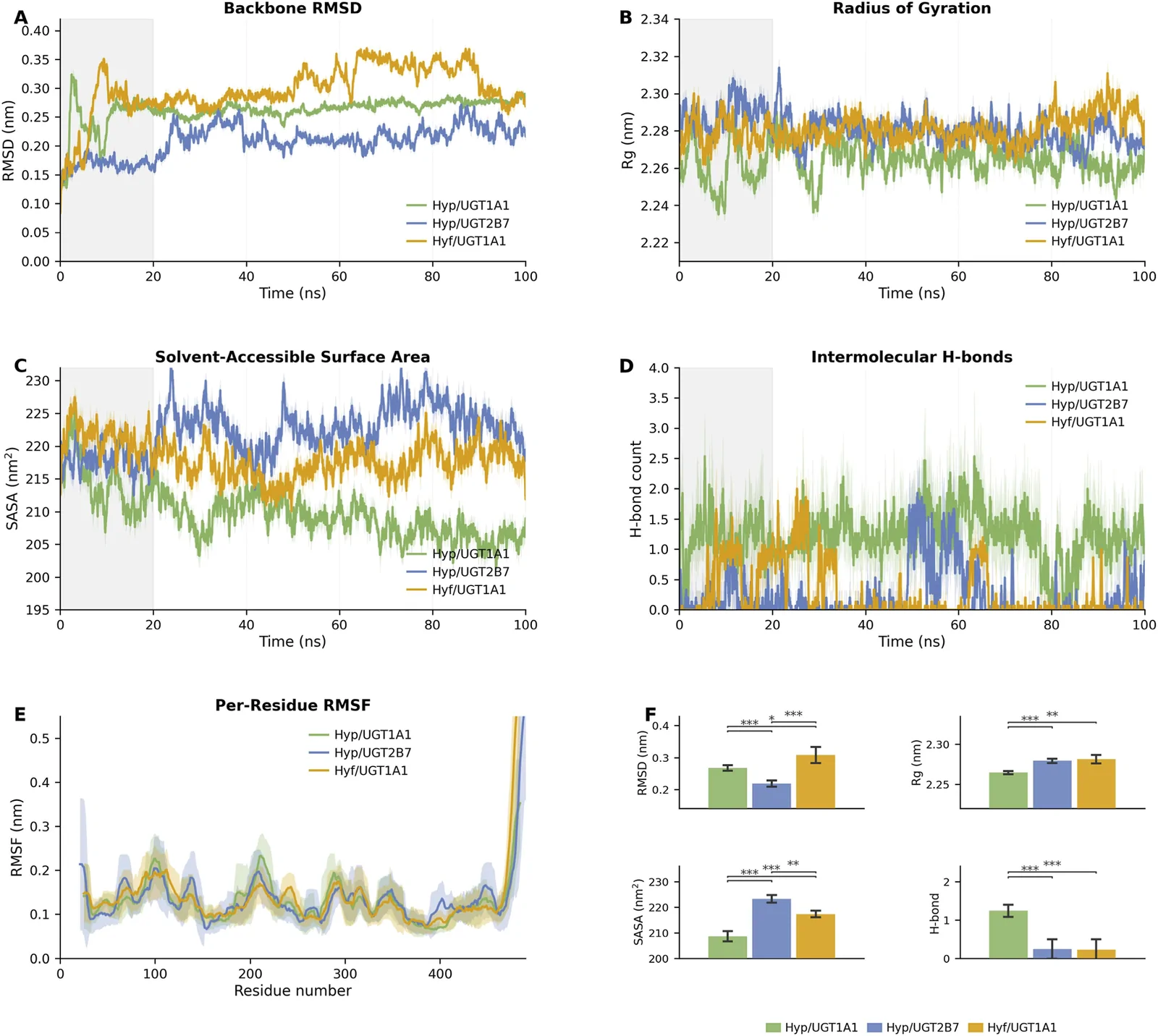
MD trajectory block analysis of three UGT–inhibitor complexes (100 ns). **(A)** Backbone RMSD (nm); **(B)** radius of gyration, Rg (nm); **(C)** solvent-accessible surface area, SASA (nm^2^); **(D)** intermolecular hydrogen-bond count; **(E)** per-residue RMSF (nm); **(F)** block-averaged comparison of RMSD, Rg, SASA, and hydrogen-bond count (mean ± inter-block SD, n = 4 × 20 ns; *p < 0.05, **p < 0.01, ***p < 0.001). The shaded 0–20 ns region in panels **(A–D)** marks the pre-equilibration period excluded from block analysis. Green: hypericin/UGT1A1; blue: hypericin/UGT2B7; orange: hyperforin/UGT1A1.

To probe the dynamic stability and structural basis of inhibition, 100 ns MD simulations were performed for three representative complexes: hypericin/UGT2B7, hypericin/UGT1A1, and hyperforin/UGT1A1 (Figure 5). Block-averaging analysis (4 × 20 ns blocks across the equilibrated 20–100 ns range) demonstrated good convergence for all three systems (coefficients of variation <10% for RMSD, <1% for Rg and SASA). Backbone RMSD values (mean ± inter-block SD) were 0.218 ± 0.010 nm for hypericin/UGT2B7, 0.267 ± 0.009 nm for hypericin/UGT1A1, and 0.308 ± 0.025 nm for hyperforin/UGT1A1; the slightly higher RMSD and SD for hyperforin/UGT1A1 reflect the flexible phloroglucinol scaffold sampling multiple sub-poses within the aglycone pocket, with a transient excursion at 60–80 ns returning to a similar steady state at 80–100 ns. The radius of gyration (Rg) remained stable across all systems (2.265–2.281 nm, CV < 0.3%), indicating that ligand binding did not induce gross conformational change.

Differences in solvent-accessible surface area (SASA) of the complex revealed three structurally distinct binding modes. Hypericin/UGT2B7 displayed the largest complex SASA (223.3 ± 1.5 nm^2^), consistent with binding at the dimer-interface surface patch identified by PLIP analysis. Hyperforin/UGT1A1 showed an intermediate SASA (217.4 ± 1.3 nm^2^), consistent with deep aglycone-pocket fitting. Hypericin/UGT1A1 showed the smallest SASA (208.6 ± 2.0 nm^2^), reflecting an extended binding mode in which the rigid polyphenolic perylenequinone scaffold occupies the aglycone pocket and extends toward the envelope helices and catalytic region.

Intermolecular hydrogen-bond counts further distinguished the three complexes. Hypericin/UGT1A1 maintained the highest average of 1.24 ± 0.16 hydrogen bonds per frame—approximately five-fold greater than hypericin/UGT2B7 (0.24 ± 0.25) and hyperforin/UGT1A1 (0.22 ± 0.27), where binding was dominated by hydrophobic and π-stacking interactions. Free-energy landscape analysis of the first two principal components revealed a single dominant low-energy basin for each complex, indicating that all three systems sampled a thermodynamically stable binding configuration over the simulation timescale. The block-averaged comparison of RMSD, Rg, SASA, and intermolecular hydrogen-bond count further confirmed statistically significant differences among the three complexes (Figure 5F).

### 3.5 MM/PBSA binding free energy decomposition of UGT–ligand complexes

**FIGURE 6:**
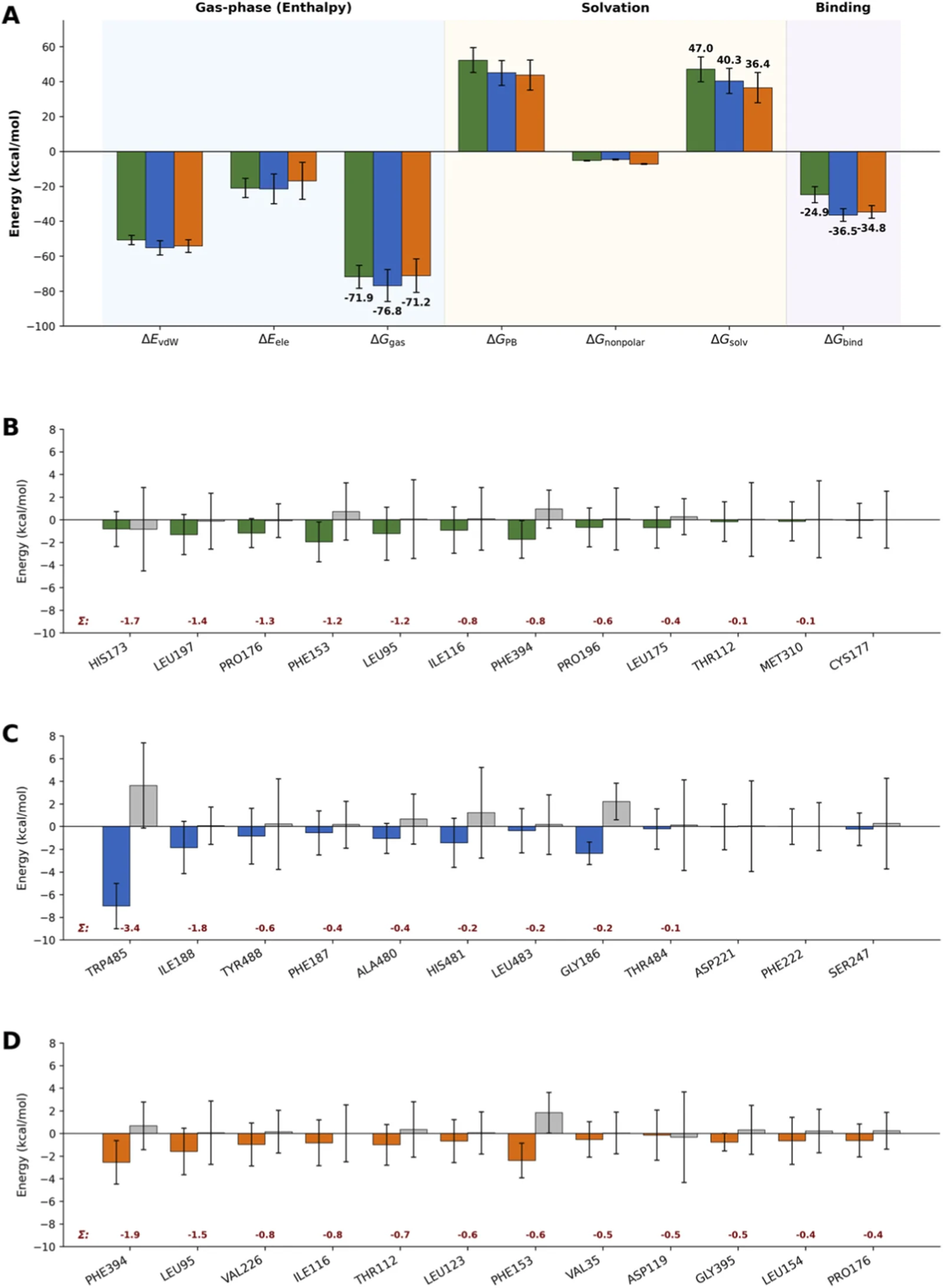
MM/PBSA binding free energy analysis of three UGT–inhibitor complexes. **(A)** Energy components for all systems; **(B–D)** per-residue decomposition for Hypericin/UGT1A1, Hypericin/UGT2B7, and Hyperforin/UGT1A1. Error bars = SD over 101 frames. Absolute ΔGbind values are enthalpy-dominated estimates; per-residue contributions used for mechanistic interpretation.

To quantify the energetic basis of the observed binding modes, MM/PBSA calculations were performed using 101 frames extracted from the equilibrated trajectory portion (20–100 ns) of each complex (Figure 6A). The total binding free energies (ΔG_bind_, mean ± SD over 101 frames) were −36.55 ± 3.65 kcal/mol for hypericin/UGT2B7, −24.87 ± 4.64 kcal/mol for hypericin/UGT1A1, and −34.80 ± 3.62 kcal/mol for hyperforin/UGT1A1. We note that these absolute values exceed the experimental ΔG derived from *K*_i_ measurements (−7.8 to −8.2 kcal/mol) due to the omission of entropy contributions, a known limitation of end-state MM/PBSA methods; we therefore emphasize component patterns and per-residue contributions for mechanistic interpretation rather than absolute affinity ranking.

All three complexes were dominated by van der Waals interactions (ΔE_vdW_ = −50.87 to −55.31 kcal/mol), reflecting extensive hydrophobic contact between the lipophilic ligands and their respective binding surfaces. The polar solvation term (ΔG_PB_ = +43.68 to +52.26 kcal/mol) imposed a substantial desolvation penalty in all systems; the electrostatic contribution (ΔG_elec_) was modest (−5.21 to −8.43 kcal/mol), and the non-polar solvation term (ΔG_SA_) was consistently small and favorable (−4.12 to −5.87 kcal/mol).

Per-residue energy decomposition (Figures 6B–D) revealed two structurally distinct UGT1A1 binding modes that nonetheless both produce noncompetitive kinetics. Hyperforin/UGT1A1 (Figure 6D) showed a localized aglycone-pocket fitting mode: the top-contributing residues—PHE394 (−2.6 kcal/mol vdW), LEU95 (−1.6), VAL226, ILE116, THR112, LEU123, PHE153 — all cluster within the N-terminal aglycone pocket, forming a compact hydrophobic envelope around the phloroglucinol scaffold. In contrast, hypericin/UGT1A1 (Figure 6B) showed an extended aglycone-pocket binding mode: top contributors HIS173 (−1.7), LEU197 (−1.4), PRO176 (−1.3), PHE153 (−1.2), LEU95 (−1.2), ILE116 (−0.8), PHE394 (−0.8), and ASP396 (−0.7) span the aglycone pocket, the connecting envelope helices, and the catalytic-domain residues. This distinction is not 'N-terminal vs. C-terminal' binding (both modes occur within the N-terminal aglycone domain) but rather the spatial extent of pocket occupation, providing a structural rationale for the shared noncompetitive mechanism arising from two different occupation patterns.

For hypericin/UGT2B7 (Figure 6C), TRP485 contributed disproportionately to binding (ΔE_vdW_ = −7.0 kcal/mol; ΔG_total_ = −3.4 kcal/mol), consistent with a π-stacking interaction between the indole side chain and hypericin’s perylenequinone scaffold at the UGT2B7 dimer-interface. Additional contributors (ILE188, TYR488, GLY186) further support a surface-patch binding mode distinct from the canonical aglycone-pocket binding observed in the UGT1A1 complexes. Across all three complexes, contributions from charged residues (Lys, Arg, Asp, Glu) showed substantially larger frame-to-frame fluctuations in their net polar component than the non-polar component, reflecting the well-recognized cancellation between gas-phase electrostatic interactions and polar solvation in the PB framework (Genheden et al., 2015); we therefore highlight the non-polar (vdW-dominated) contributions as the structurally robust binding hotspots in Figures 6B–D.

### 3.6 IVIVE prediction of DDI risk

The predicted pharmacokinetic parameters and calculated risk ratios are presented in Table 2. For hypericin, *R*_1_ values for all tested UGT isoforms were 1.00, indicating negligible risk of systemic DDIs. The *R*_1,gut_ values ranged from 2.14 to 4.05, predicting a low risk of clinically significant intestinal DDIs.

**TABLE 2:** IVIVE prediction of clinical DDI risk for hypericin and hyperforin based on the FDA 2020 static model.

Compound	UGTs	*K*ᵢ,_u_ (μM)	[I]_gut_ (μM)	C_max_ (μM)	*f*u	[I]_max,u_ (μM)	*R*_1_	*R*_1_,_gut_
Hypericin	UGT1A1	2.80	6.90	0.03	0.01	0.0003	1.00	3.46
​	UGT1A6	3.35	6.90	0.03	0.01	0.0003	1.00	3.06
​	UGT1A9	3.55	6.90	0.03	0.01	0.0003	1.00	2.94
​	UGT2B4	3.47	6.90	0.03	0.01	0.0003	1.00	2.99
​	UGT2B7	6.04	6.90	0.03	0.01	0.0003	1.00	2.14
​	UGT2B15	2.26	6.90	0.03	0.01	0.0003	1.00	4.05
Hyperforin	UGT1A1	1.36	93.14	0.24	0.02	0.0048	1.00	69.49*
​	UGT2B7	6.24	93.14	0.24	0.02	0.0048	1.00	15.93*

*f*_u_: unbound fraction in plasma (hypericin = 0.01; hyperforin = 0.02); *R*_1_: hepatic risk ratio; *R*_1_,_gut_: intestinal risk ratio. FDA, thresholds: *R*_1_ ≥ 1.02 (systemic DDI, concern); *R*_1_,_gut_ ≥ 11 (intestinal DDI, concern). *K*ᵢ,_u_ = *K*ᵢ × *f*_u,inc_ (unbound inhibition constant). [I]_max,u_ = C_max_ × *f*_u_. [I]_gut_ = Dose/250 mL (complete dissolution assumed). C_max_ for hypericin: ∼16 ng/mL (0.03 μM); for hyperforin: ∼128 ng/mL (0.24 μM). *R_1_,_gut_ exceeds FDA, high-risk threshold of 11.

In marked contrast, hyperforin demonstrated a pronounced intestinal DDI risk. The systemic *R*_1_ was 1.00 (low risk), but the intestinal risk was exceptionally high. With [I]g_ut_ calculated at 93.14 μM, the *R*_1,gut_ values for UGT1A1 (69.49) and UGT2B7 (15.93) exceeded the FDA high-risk threshold of 11 under the complete-dissolution assumption (a theoretical upper bound).

Sensitivity analysis incorporating the reported variability in commercial SJW hyperforin dosages (9–17 mg/dose) and C_max_ values (approximately 80–300 ng/mL) confirmed the robustness of this prediction. For UGT1A1, the *R*_1,gut_ range was approximately 50–94; for UGT2B7, approximately 12–21. Even at the lowest dosage range, the intestinal risk ratios remained above the FDA threshold. The additional AUC ratio-based IVIVE model (Supplementary Figure S4) supported the conclusion that systemic risk is moderate while intestinal risk is high.

## 4 Discussion

### 4.1 Summary of key findings

The co-administration of herbal medicines with conventional drugs is a growing public health concern, primarily due to the risk of unforeseen DDIs that can alter drug efficacy and safety profiles (Chen et al., 2012; Jeong et al., 2025). St. John’s Wort is a prime example, with a well-documented history of clinically significant CYP-mediated and P-gp-mediated interactions (Nicolussi et al., 2020). However, the impact of SJW on the UGT enzyme family has remained a critical knowledge gap. The present study provides the first comprehensive investigation of this question, demonstrating that both hypericin and hyperforin are potent broad-spectrum UGT inhibitors and that hyperforin poses a high and robust intestinal DDI risk that substantially exceeds the FDA threshold across all tested dosage scenarios.

### 4.2 Comparison with existing literature on natural product UGT inhibitors

Our *in vitro* assays establish both compounds as potent, broad-spectrum UGT inhibitors. Docking provided a hypothesis-generating structural context (Supplementary Figure S2): hypericin engaged all six isoforms favourably (ΔG –10.2 to −11.3 kcal/mol, most favourable for UGT2B7), while hyperforin scored better for UGT1A1 (−8.4 kcal/mol) than UGT2B7 (−7.1 kcal/mol), directionally consistent with the measured *K*_i_ (1.46 vs. 6.70 μM). These poses are qualitative only; a quantitative ΔG–*K*_i_ correlation is not implied, and co-crystallography or cryo-EM is required for validation.

Although the DDI narrative centres on hyperforin, hypericin was also a potent broad-spectrum inhibitor (six isoforms, *K*_i_ 2.28–6.08 μM) with distinct binding modes—an extended aglycone-pocket occupancy in UGT1A1 and a dimer-interface π-stacking interaction at UGT2B7 (TRP485). Its systemic and intestinal DDI risk is nonetheless low because its [I]_gut_ and [I]_max,u_ are far lower than those of hyperforin, which explains why hyperforin, despite comparable *in vitro* potency, dominates the predicted clinical risk.

In potency terms, hyperforin’s UGT1A1 *K*_i_ (1.46 μM) is comparable to quercetin (2.18 μM (Zhang et al., 2021)) and to atazanavir (1.9 μM (Zhang et al., 2005)), whose mixed-type UGT1A1 inhibition is among the best-documented clinical drug–UGT interactions. What distinguishes SJW from dietary polyphenols is the mode of exposure: polyphenols arrive intermittently with food, whereas SJW is taken daily at standardised doses, sustaining inhibitor concentrations at the intestinal mucosa for weeks to months. Under such chronic exposure, a low-micromolar *K*_i_ becomes a clinically meaningful DDI liability rather than an *in vitro* curiosity.

### 4.3 IVIVE-derived DDI risk and its mechanistic basis

IVIVE using the FDA 2020 static model revealed a sharp divergence between systemic and intestinal risk. Assuming complete dissolution ([I]_gut_ = Dose/250 mL = 93 μM for a single 12.5 mg hyperforin dose; baseline scenario, Table 3), *R*_1,gut_ reached 69.49 (UGT1A1) and 15.93 (UGT2B7), both exceeding the FDA threshold of 11, whereas systemic *R*_1_ remained near 1.0.

**TABLE 3:** Sensitivity analysis of hyperforin intestinal DDI risk (*R*_1_,_gut_) across the range of commercial SJW product dosages and reported C_max_ values.

Scenario	Dose (mg)	C_max_ (ng/mL)	[I]_gut_ (μM)	*K*ᵢ,_u_ (μM)	*R*_1_,_gut_ UGT1A1	*R*_1_,_gut_ UGT2B7
Low dose (minimum)	9	∼80	67.06	1.36	∼50.3	∼11.7
Baseline (Schulz (Schulz et al., 2005))	12.5	∼128	93.14	1.36	69.49	15.93
High dose (maximum)	17	∼300	126.68	1.36	∼94.1	∼21.3
FDA high-risk threshold	—	—	—	—	≥11	≥11

*R*_1_,_gut_ = 1 + [I]_gut_/*K*ᵢ_,u_. [I]gut scaled proportionally from baseline scenario. *K*ᵢ,_u_: UGT1A1 = 1.36 μM; UGT2B7 = 6.24 μM. C_max_ ranges from Schulz et al. (Schulz et al., 2005), Biber et al. (Biber et al., 1998), and Gödtel-Armbrust et al. (Gödtel-Armbrust et al., 2007). FDA, high-risk threshold: *R*_1_,_gut_ ≥ 11.

This theoretical ceiling, however, rests on two assumptions—complete aqueous dissolution and gastrointestinal stability—that the literature does not support: hyperforin dissolves from commercial formulations at only about 2% (Jürgenliemk and Nahrstedt, 2003) and degrades under gastric acid (Ang et al., 2004). Incorporating these constraints, the realistic luminal [I]_gut_ falls to roughly 1.3–2.5 μM, giving corrected *R*_1,gut_ values of about 2.0–2.9 (UGT1A1) and 1.2–1.4 (UGT2B7)—still above the systemic warning threshold but below the stricter intestinal criterion. Hyperforin is therefore best characterised as a potential, rather than definitive, intestinal UGT inhibitor whose real-world impact depends on the dissolution and stability profile of the specific SJW preparation administered. Nevertheless, patients receiving narrow-therapeutic-index UGT substrates (e.g., irinotecan, morphine) alongside high-bioavailability SJW formulations should be monitored, as the actual inhibitory exposure will vary substantially with product-specific dissolution profiles.

Kinetically, the noncompetitive inhibition of UGT1A1 and UGT2B7 by hyperforin—both central to the intestinal prediction—makes the FDA *R*_1_ formula directly applicable without α-dependent correction (FDA, 2020). For the two hypericin mixed-inhibition systems (UGT2B4, α = 3.09; UGT2B7, α = 0.13), *R*_1_ was estimated using the linear-region approximation; sensitivity analysis (Supplementary Table S1) shows that this approximation is accurate at low victim-drug concentrations but progressively over-estimates (UGT2B4) or under-estimates (UGT2B7) *R*_1_ at supra-*K*_m_ concentrations. Because the principal intestinal-DDI conclusion rests on the noncompetitive inhibition of UGT1A1 and UGT2B7 by hyperforin—for which the FDA *R*_1_ formula is exact—this approximation does not affect the main findings.

These findings should be placed within the established SJW–DDI framework. The clinical interaction profile of SJW is largely attributed to hyperforin-mediated activation of the pregnane X receptor (PXR) and consequent induction of CYP3A4 and P-glycoprotein, which predominates during chronic dosing and underlies the best-documented interactions, including those with cyclosporine, indinavir, and oral contraceptives (Nicolussi et al., 2020; Jiang et al., 2022). The acute, direct UGT inhibition described here is a mechanistically distinct, complementary pathway—most relevant during single-dose, first-dose, or short-term exposure, before the 7–14 days lag required for clinically meaningful CYP induction (Nicolussi et al., 2020). Notably, several established SJW victim drugs are also UGT substrates (ethinylestradiol via UGT1A1/UGT2B7; irinotecan SN-38 via UGT1A1), and a recent comprehensive review (Miners et al., 2023) documents that UGT-mediated DDIs are more clinically relevant than historically assumed. Acute UGT inhibition should therefore be regarded as complementary to, not in competition with, the chronic induction pathway.

### 4.4 Clinical implications

The clinical relevance of intestinal UGT inhibition is clearest for drugs undergoing extensive first-pass glucuronidation. Irinotecan’s active metabolite SN-38 is detoxified almost exclusively by UGT1A1; its inhibition could sharply increase SN-38 exposure and the risk of severe neutropenia and diarrhoea (Sun et al., 2020; Su et al., 2023). UGT2B7 is the primary route for morphine (Stone et al., 2003; Ohno et al., 2008) and zidovudine (Barbier et al., 2000) glucuronidation, both narrow-therapeutic-index drugs for which impaired clearance could precipitate respiratory depression or haematological toxicity. Although the theoretical *R*_1,gut_ for UGT1A1 (about 50 at the 9 mg dose and 94 at 17 mg; Table 3) overstates real-world risk, the roughly 62-fold spread in hyperforin content across commercial SJW products (Gödtel-Armbrust et al., 2007) leaves patients and prescribers unable to gauge DDI risk from the label. This product-to-product variability, together with persistently elevated risk ratios across all dose scenarios, argues for tighter standardisation and regulatory scrutiny of SJW supplements (Nicolussi et al., 2020; Chen et al., 2012).

### 4.5 Limitations

Several limitations should be considered. First, *f*_u,inc_ was estimated from the Austin et al. empirical equation (Austin et al., 2002) rather than measured directly; equilibrium dialysis would be more accurate, particularly for the highly lipophilic hyperforin. Second, the structural work relied on AlphaFold2 full-length monomer models, as no complete human UGT crystal structure exists—only isolated N-terminal fragments and the UGT2B7 C-terminal domain have been solved (Miley et al., 2007; Locuson and Tracy, 2007). Although mean pLDDT exceeded 90 and binding-pocket confidence was high, we mitigated this by cross-docking atazanavir, a clinically characterised UGT1A1 inhibitor, which reproduced the expected N-terminal aglycone-pocket mode and overlapped the hyperforin MM/PBSA signature. Third, MM/PBSA absolute ΔG_bind_ values exceeded experimental *K*_i_-derived ΔG because explicit entropy (−TΔS) was omitted, normal-mode analysis being prohibitive for proteins larger than 500 residues (Genheden et al., 2015); per-residue contributions and energy-component patterns were therefore used for mechanistic interpretation rather than absolute affinity ranking. Fourth, a single 100 ns trajectory was simulated per system—block averaging confirmed intra-trajectory convergence (CV < 10%), but independent replicates would better establish binding-mode reproducibility—and MD was limited to three of the eight inhibition systems.

Fifth, hyperforin was characterised in only two isoforms (UGT1A1 and UGT2B7), so the proposed ligand-class behaviour requires confirmation in additional isoforms. Sixth, the mixed-inhibition systems used the linear-region *R*_1_ approximation, justified when victim-drug concentrations are well below *K*_m_ but warranting the full α-dependent formula for high-concentration predictions. Seventh, the *R*_1_ calculations assume a fraction metabolised (*f*_m_) approaching unity, a theoretical maximum; because most glucuronidated drugs are cleared by parallel UGT and CYP pathways, where *f*_m_ < 1 (for example, UGT1A1 accounts for roughly 0.5–0.7 of SN-38 clearance) the actual AUC change will be proportionally smaller, and definitive victim-drug predictions will require substrate-specific PBPK modelling. Finally, the *in vitro* characterisation used 4-methylumbelliferone (4-MU) as a universal probe rather than isoform-specific substrates or human intestinal microsomes (HIMs). This choice—consistent with FDA 2020 guidance (FDA, 2020) and widely adopted (Uchaipichat et al., 2004; Zhang et al., 2021)—enabled the systematic cross-isoform comparison central to the broad-spectrum profile reported here; the selective UGT2B15 probe S-oxazepam is a controlled substance legally inaccessible in our setting, substrate-dependent inhibition modality is documented for UGTs (Uchaipichat et al., 2004; Uchaipichat et al., 2008), and UGT1A1 and UGT2B7 are both highly expressed in intestine with liver-comparable kinetics (Rowland et al., 2013; Miners et al., 2023), so the FDA intestinal model applies without direct HIM measurement. Although absolute *K*_i_ values obtained with 4-MU may differ from those measured with isoform-specific substrates, the noncompetitive mechanism central to our conclusions was determined by full kinetic analysis (Lineweaver–Burk and secondary-slope replots) and is therefore independent of the probe substrate; isoform-specific-substrate validation (β-estradiol for UGT1A1, AZT for UGT2B7) would refine absolute *K*i values but would not alter the broad-spectrum profile, the noncompetitive mechanism, or the high intestinal *R*_1,gut_ prediction. Only reversible inhibition was evaluated; given hyperforin’s CYP-inducing activity, time-dependent (mechanism-based) inhibition and transcriptional UGT regulation warrant future study, and direct HIM and isoform-specific validation are noted in Future Directions (Section 4.6).

Recombinant single-enzyme systems were chosen to isolate individual isoform contributions and enable mechanistic attribution and uniform cross-isoform comparison that pooled microsomes cannot provide. Inhibitory potency in recombinant systems may nonetheless differ from that in human liver or intestinal microsomes owing to differences in membrane lipid environment, relative enzyme abundance, protein–protein interactions, and competing metabolic pathways, which may either over- or under-estimate *in vivo* inhibition. Confirmation in human liver microsomes, human intestinal microsomes, and hepatocyte systems is therefore warranted (Section 4.6).

### 4.6 Future research directions

Future research should prioritize: (1) experimental determination of *f*_u,inc_ for hyperforin and hypericin using equilibrium dialysis; (2) assessment of time-dependent inhibition kinetics for both compounds; (3) as a priority, confirmatory determination of isoform-specific Ki values using isoform-selective substrates (β-estradiol for UGT1A1, AZT for UGT2B7), to test the substrate-dependence of the absolute *K*_i_ values reported here; (4) PBPK modeling to refine intestinal DDI predictions; and (5) clinical pharmacokinetic studies to validate the predicted interactions, particularly for irinotecan-SJW and morphine-SJW combinations in relevant patient populations. (6) validation using crude/whole SJW extracts containing all constituents, to capture possible synergism and potentiation and the combined inhibition and induction of UGTs and P-glycoprotein under real-world conditions of use, thereby closing the gap between single-compound mechanistic data and clinical application.

## 5 Conclusion

Taken together, the present findings establish that the principal bioactive constituents of SJW—hypericin and hyperforin—are potent inhibitors across the UGT enzyme family. The systemic DDI risk they pose appears modest in conventional IVIVE calculations, yet the intestinal risk, particularly that of hyperforin against UGT1A1 and UGT2B7, warrants serious clinical attention. This concern is compounded by the wide and poorly controlled variation in bioactive content among commercial SJW products, which makes reliable risk prediction from labeling impossible. Greater vigilance is warranted when SJW is co-prescribed with drugs whose clearance depends substantially on intestinal glucuronidation, and these data provide a mechanistic basis to inform such clinical decision-making as well as to motivate future regulatory action and clinical pharmacokinetic studies.

## Data Availability

The original contributions presented in the study are included in the article/Supplementary Material, further inquiries can be directed to the corresponding authors.
