# Supplementary material for: Hypericin and hyperforin from St. John’s Wort are potent intestinal UGT inhibitors with distinct binding modes: mechanistic and quantitative prediction of clinical drug–drug interaction risk
Source: Front Pharmacol. 2026 Sep 10;17:1899795. doi: 10.3389/fphar.2026.1899795 (PMC13601336; doi:10.3389/fphar.2026.1899795)
Supplement: Supplementary file 1 [file DataSheet1.pdf]

Supplementary Materials

Hypericin and Hyperforin from St. John's Wort Are Potent Intestinal UGT Inhibitors with Distinct Binding Modes: Mechanistic and Quantitative Prediction of Clinical Drug–Drug Interaction Risk

Jinqian Chen †, Yanlong Zhang †, Xixi Zhao, Yating Cui, Tong Xu, Lingxia Liu, Zhenyu Zhao *, and Xichuan Li *

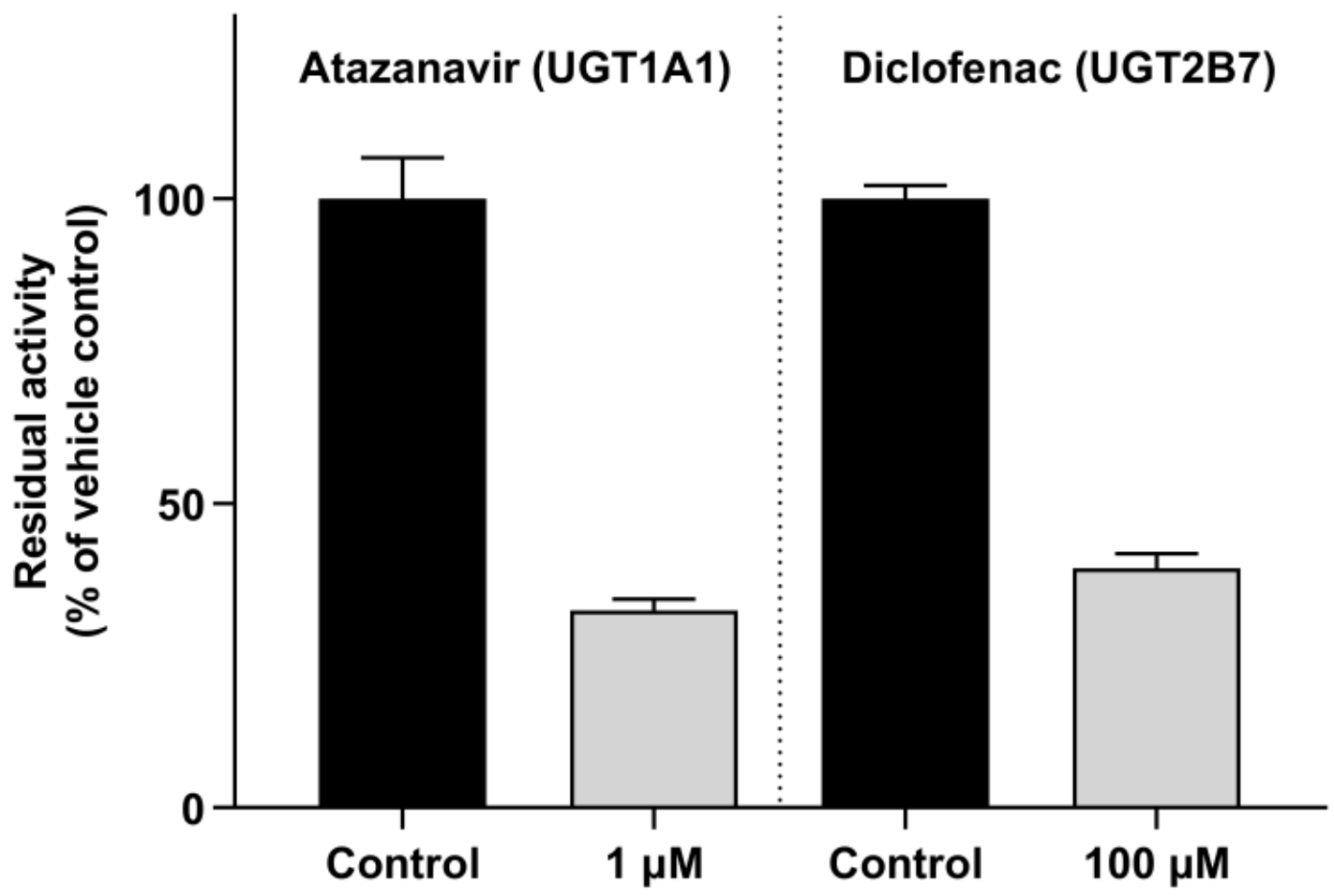

Figure S1. Positive-control inhibition confirming the functional activity and inhibitability of the recombinant enzyme preparations. Residual 4-MU glucuronidation activity (% of vehicle control) is shown for atazanavir (UGT1A1) and diclofenac (UGT2B7). Atazanavir inhibited UGT1A1 by 67.6% at 1 μM (residual 32.4 ± 1.9%) and completely (100%) at 100 μM; the 100 μM condition (complete inhibition) is omitted from the plot. Diclofenac inhibited UGT2B7 by 60.6% at 100 μM (residual 39.4 ± 2.4%). Bars are mean ± S.D. of three independent replicates (n = 3). Diclofenac was used as a positive-control inhibitor rather than an isoform-selective probe.

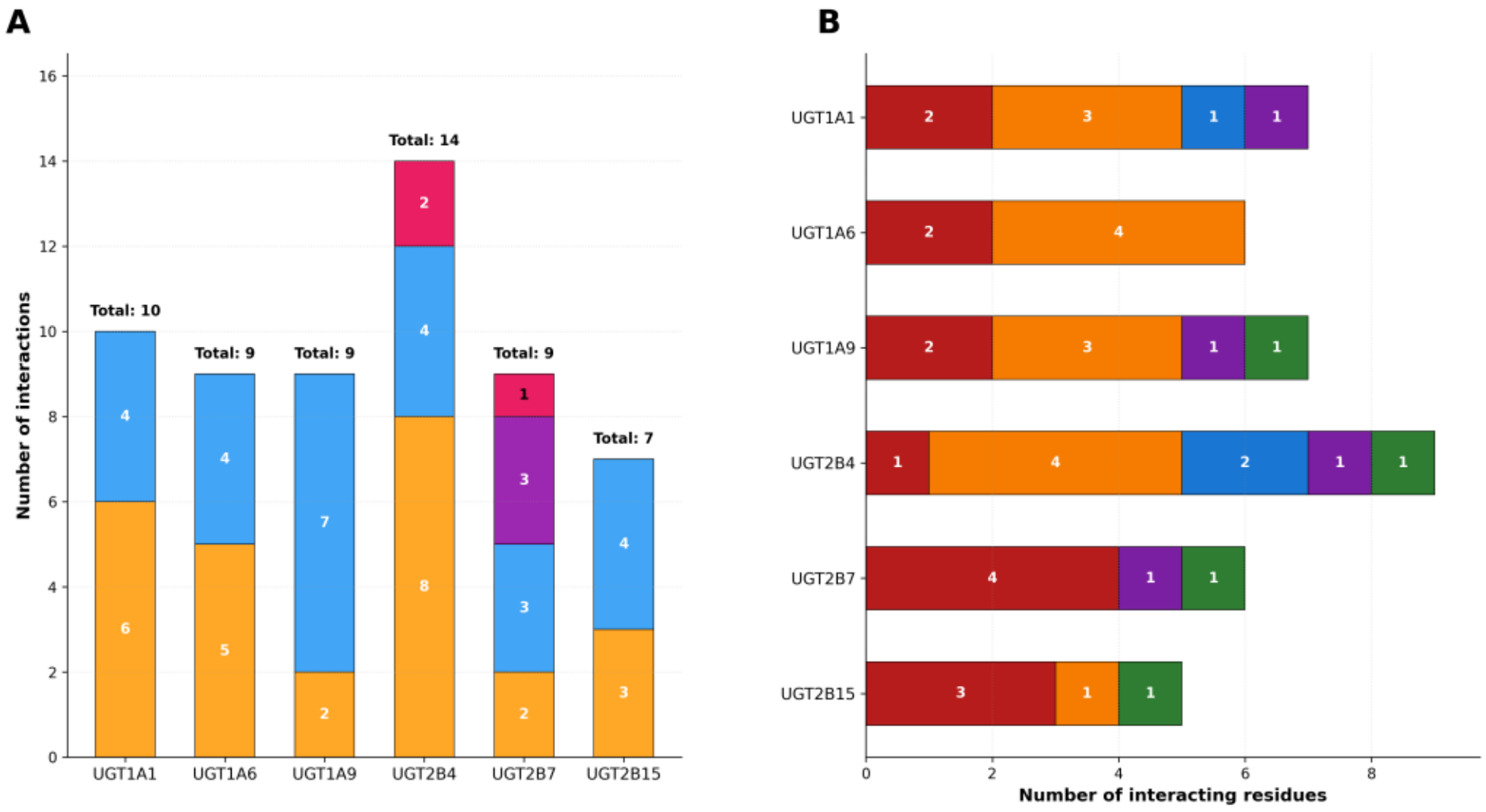

Figure S2. Cross-docking validation with atazanavir and hypericin docking poses across six UGT isoforms. (A) Atazanavir top-ranked pose in AlphaFold2 UGT1A1 (ΔG_{dock} = −9.3 kcal/mol), reproducing expected aglycone-pocket contacts and validating the pipeline. (B–G) Hypericin docking poses in UGT1A1, UGT1A6, UGT1A9, UGT2B4, UGT2B7, UGT2B15. Key PLIP contacts shown; ΔG_{dock} annotated. Hypothesis-generating only.

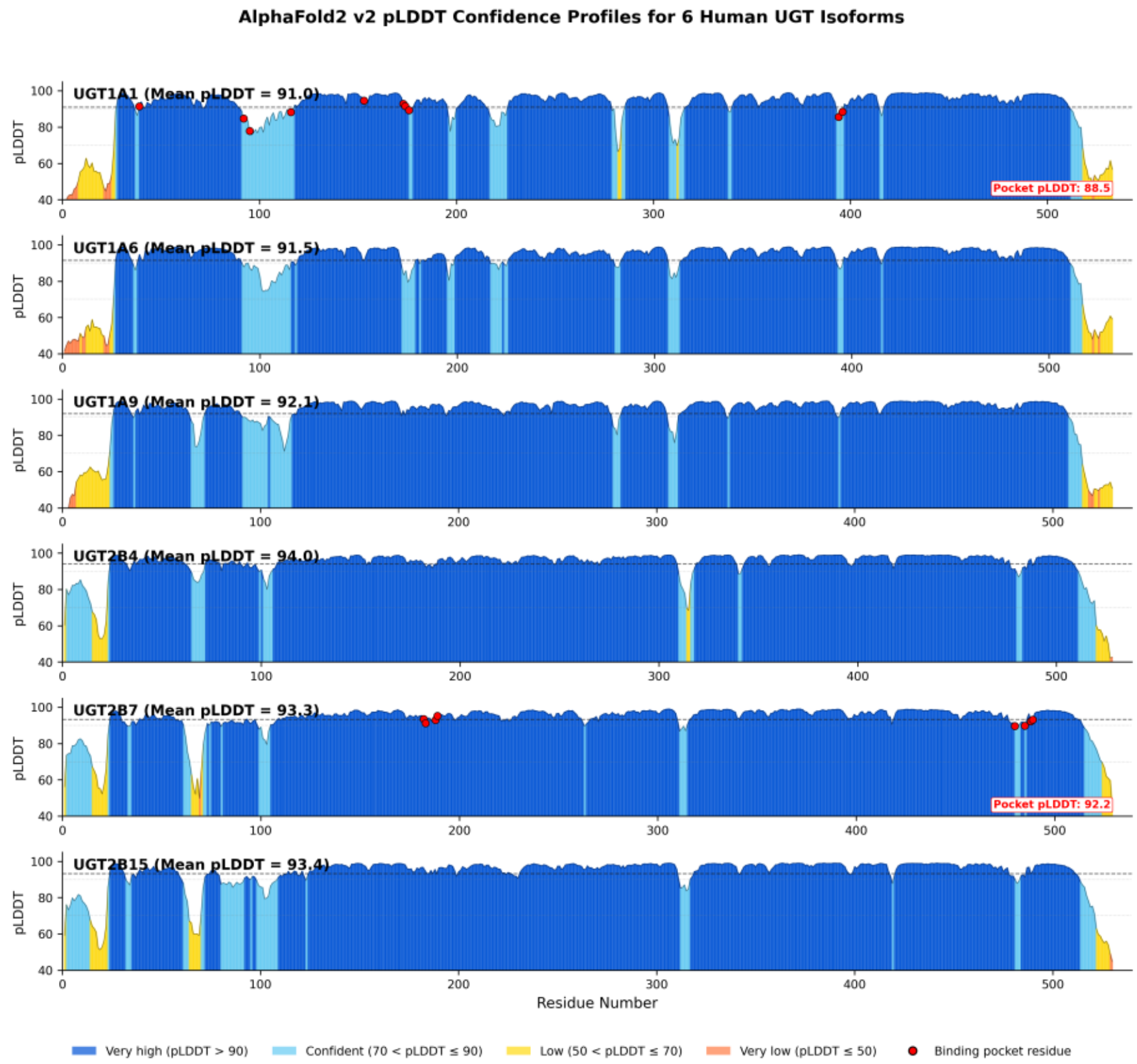

Figure S3. AlphaFold2 v2 pLDDT confidence profiles for six human UGT isoforms. Dark blue: pLDDT > 90; light blue: 70–90; yellow: 50–70; orange-red: ≤50. Red circles: binding-pocket residues (UGT1A1 and UGT2B7). Dashed line: pLDDT = 90. Full statistics in Table S2.

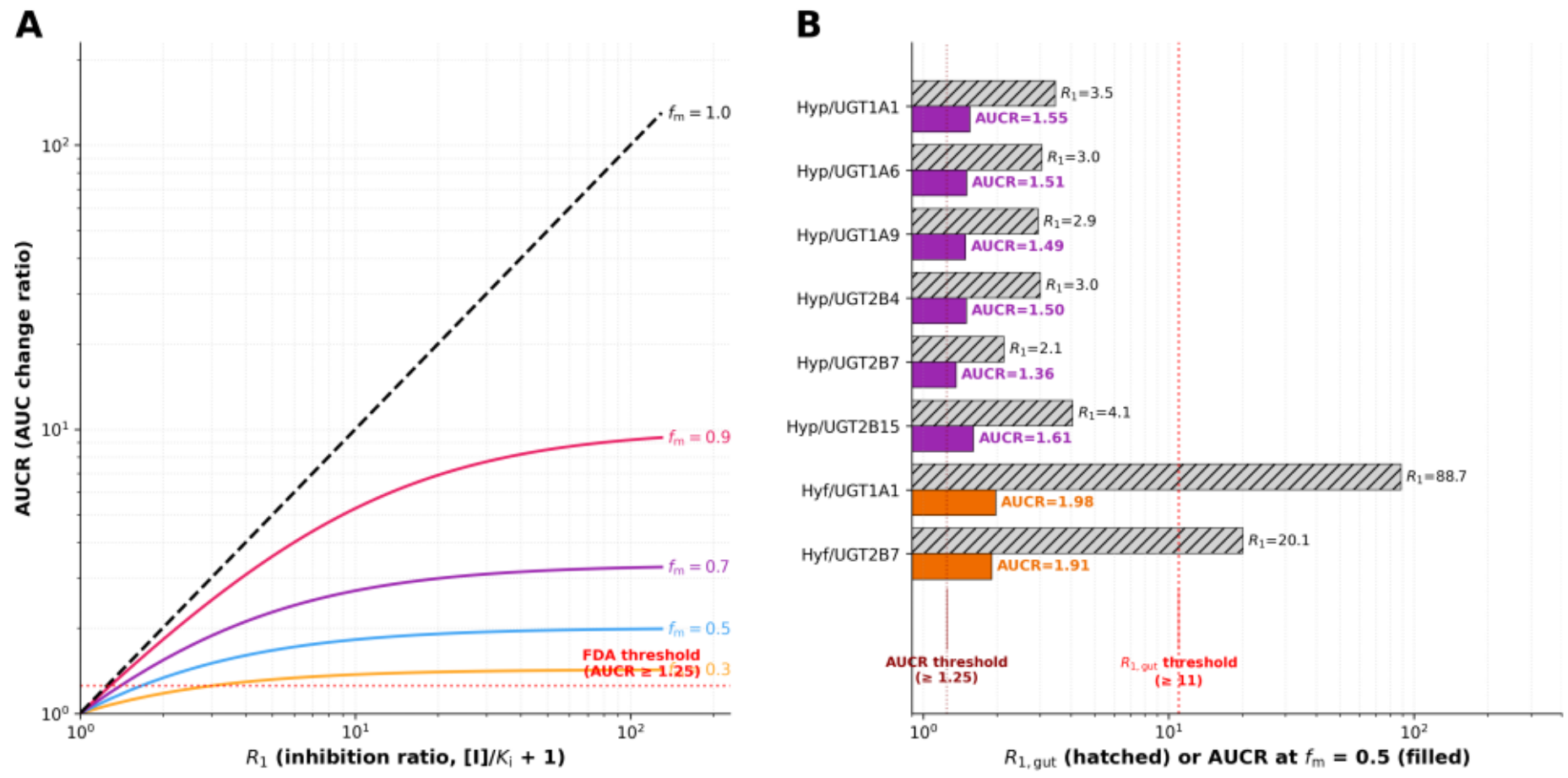

Figure S4. AUC ratio (AUCR)-based IVIVE model for clinical DDI risk prediction. (A) AUCR vs. R_1 for $f_m = 0.3, 0.5, 0.7, 0.9, 1.0$; FDA threshold (AUCR ≥ 1.25) shown. (B) Forest plot of $R_{1,gut}$ (hatched) and AUCR at $f_m = 0.5$ (filled) for all pairs. Hyperforin/UGT1A1 and UGT2B7 exceed both thresholds.

Supplementary Tables

Table S1. Sensitivity analysis of R_1 deviations across [S]/K_m ratios for the two mixed-inhibition systems.

	UGT2B4 (Hypericin, α = 3.09)			UGT2B7 (Hypericin, α = 0.13)		
**[S]/K_m**	**$R_{1,lin}$**	**$R_{1,mix}$**	**Δ (%)**	**$R_{1,lin}$**	**$R_{1,mix}$**	**Δ (%)**
0.1	3.012	2.888	−4.1	2.139	2.831	+32.4
0.3	3.012	2.698	−10.4	2.139	3.897	+82.2
1.0	3.012	2.332	−22.6	2.139	5.949	+178.2†
3.0	3.012	1.991	−33.9	2.139	7.854	+267.2†
10.0	3.012	1.775	−41.1	2.139	9.066	+323.9†

$R_{1,lin} = 1 + [I]/K_i$(FDA linear approximation). $R_{1,mix}$: exact mixed-inhibition formula. Δ (%): percentage deviation of $R_{1,lin}$ from $R_{1,mix}$. † |Δ| > 100%, indicating the linear approximation substantially underestimates R_1 at supra-K_m substrate concentrations for the UGT2B7 system (α = 0.13). Input: $[I]_{gut}$ = 6.90 μM (hypericin, complete dissolution).

Table S2. AlphaFold2 pLDDT confidence statistics for the six UGT isoforms used in molecular docking and MD simulations.

UGT	UniProt	Length (aa)	Mean pLDDT	Median pLDDT	% > 70	% > 90	Pocket pLDDT
UGT1A1	P22309	533	91.0	95.7	91.6	78.2	88.5
UGT1A6	P19224	532	91.5	96.2	92.3	79.5	N/A
UGT1A9	O60656	530	92.1	96.2	92.6	82.8	N/A
UGT2B4	P06133	528	94.0	96.8	96.2	87.3	N/A
UGT2B7	P16662	529	93.3	95.9	96.0	87.0	92.2

UGT2B15	P54855	530	93.4	96.9	95.3	83.4	N/A
Range	—	528–533	91.0–94.0	95.7–96.9	91.6–96.2	78.2–87.3	88.5–92.2

Pocket pLDDT: mean pLDDT of residues within 5 Å of the docked ligand (UGT1A1 and UGT2B7 only; MD/MM–PBSA performed for these two isoforms). N/A: not applicable. All structures retrieved from the AlphaFold Protein Structure Database [22].

Table S3. Molecular docking binding free energies and key protein–ligand interactions (PLIP) for hypericin and hyperforin across the eight docked UGT–inhibitor complexes.

Complex	ΔGdock (kcal/mol)	Key interacting residues (PLIP)
Hypericin–UGT1A1	−10.7	Hydrophobic: ILE116, PHE153, ALA174, PRO176; H-bond: SER38, HIS39, ALA174, ASP396
Hypericin–UGT1A6	−10.2	Hydrophobic: PHE259, VAL272, PHE273, LEU456; H-bond: MET271, VAL451
Hypericin–UGT1A9	−10.9	Hydrophobic: VAL258, PRO451; H-bond: ASP256, PHE257, VAL258, HIS444, ARG447, VAL449
Hypericin–UGT2B4	−11.1	Hydrophobic: LYS95, ALA98, GLU99, VAL313, LEU395, PHE417; H-bond: LYS95, MET312, ASN315, SER317; π-cation: LYS95
Hypericin–UGT2B7	−11.3	Hydrophobic: LYS182, TYR488; H-bond: PHE189, ASP221, TYR488; π-stacking: HIS183; π-cation: HIS489
Hypericin–UGT2B15	−10.2	Hydrophobic: LYS183, ALA481, TYR489; H-bond: PHE188, HIS482, TYR489
Hyperforin–UGT1A1	−8.4	Hydrophobic: PHE92, LEU95, THR112, LYS115, ILE116, PHE153, PRO176, LEU222, PHE394; H-bond: ASP119
Hyperforin–UGT2B7	−7.1	Hydrophobic: MET37, TRP356, ILE357, PRO358, GLN359, ASN360; H-bond: ASN360

ΔGdock: top-ranked AutoDock Vina binding score. Non-covalent contacts identified with PLIP v2.4.0 [27]; hydrophobic, hydrogen-bond (H-bond), π-stacking, and π-cation interactions are listed. Docking poses are hypothesis-generating (see Figure S2).
